# HIV-1 Nef activates proviral DNA transcription by recruiting Src kinase to phosphorylate host protein Nef-associated factor 1 to compromise its viral restrictive function

**DOI:** 10.1128/jvi.00280-25

**Published:** 2025-04-24

**Authors:** Tian-Jiao Fan, Chengzuo Xie, Lisha Li, Xia Jin, Jie Cui, Jian-Hua Wang

**Affiliations:** 1 Pingyuan Laboratory, State Key Laboratory of Antiviral Drugs, Henan Normal University 26468, Xinxiang, China; 2 Shanghai Sci-Tech Inno Center for Infection & Immunity, Shanghai, China; 3 Guangzhou Institutes of Biomedicine and Health, Chinese Academy of Sciences 74627, Guangzhou, China; 4 Graduate School of Guangzhou Medical University 66519, Guangzhou, China; University Hospital Tübingen, Tübingen, Germany

**Keywords:** HIV, Nef, Nef-associated factor 1, proviral transcription

## Abstract

HIV-1 accessory protein Nef is a multifunctional pathogenic factor that mediates immune evasion, enhances virion infectivity, antagonizes host restrictive factors, and promotes viral dissemination. However, the modulation of Nef on proviral DNA transcription of latently infected viruses is not well understood. In this study, we found that Nef activated HIV-1 proviral DNA transcription by recruiting Src Family Kinases (SFKs) member Src to stimulate the downstream PI3K/AKT/mTOCR1/CDK9 cellular pathway, and that Naf1 (Nef-associated factor 1), a host protein that is known to suppress HIV-1 transcription, was required for this function of Nef. This seemingly contradictory interplay between Nef and Naf1 was investigated. Naf1 was a repressor of the PI3K/AKT/mTOCR1/CDK9 cellular pathway, but in the presence of Nef, Naf1 was phosphorylated at the Tyrosine-552 by Nef-recruited Src, consequently converting its normal restrictive role to coordinate with Nef to activate proviral DNA transcription. These findings reveal a mechanism by which Nef activates HIV-1 proviral DNA transcription and discover the dual function of Naf1 protein in regulating HIV infection, depending on its phosphorylation status. This study reports a new interaction mode between host factors and viral proteins in regulating HIV-1 replication.

**IMPORTANCE:**

HIV-1 accessory protein Nef is a multifunctional pathogenic factor; however, the modulation of Nef on proviral DNA transcription of latently infected virus is not well understood. This study demonstrates Nef's role in activating HIV-1 proviral DNA transcription and uncovers the underlying cellular mechanism. Nef recruits Src kinase to phosphorylate Naf1, and the phosphorylation of Naf1 converts its normal restrictive role to coordinate with Nef to activate proviral DNA transcription by stimulating the downstream PI3K/AKT/mTOCR1/CDK9 cellular pathway. These findings also report a new interaction mode between host factors and viral proteins in regulating HIV-1 replication.

## INTRODUCTION

HIV-1 accessory protein Nef is a crucial pathogenic factor. It manipulates various cellular machineries to establish a favorable environment for viral replication, immune evasion, and viral spread (1–9). The well-described function of Nef includes its downregulation of cell surface expression of CD4, CD28, MHC-1, HLA, CXCR4, CCR5, dendritic cell-specific intercellular adhesion molecule-3-grabbing non-integrin (DC-SIGN), and the restrictive factors serine incorporator-3 and -5 (SERINC-3, -5) (1, 2, 10–18). Nef can also inhibit immunoglobulin class switching by penetrating B cells (19, 20), and modulating T cell activation (21), to promote viral infection, transmission, and immune escape. The interactions between Nef proteins of HIV-1 or simian immunodeficiency virus (SIV) and other viral/host proteins are required for efficient viral replication and pathogenesis (22–24).

Nef does not harbor catalytic activity, but it exerts functions by recruiting multiple host proteins to activate cellular signaling cascades. Nef protein from all major HIV-1 clades could activate Src Family Kinases (SFKs) to enhance viral replication (25). SFKs are engaged in Nef-mediated functions. The binding with SFK member Hck (hemopoietic cell kinase) induces conformational changes in the Nef protein to expose its residues critical for interaction with AP1 and MHC-1 molecules (26, 27). The ^72^PXXP^75^ motif of Nef forms a binding region for the SH3 domain of SFKs, and this interaction of Nef with SFKs could further recruit ZAP-70 (Zeta-Chain Associated Protein Kinase, 70kD) to activate phosphatidylinositol-3 kinase (PI3K) and the downstream ARF6 (ADP ribosylation factor 6), leading to MHC endocytosis and the delayed recycling to the plasma membrane (28, 29). Inhibition of the activation of Hck results in a marked reduction of Nef-enhanced HIV-1 replication (25). The serine/threonine kinase Pak2 (p21-activated kinase 2) can also be activated by Nef. Pak2 potentially functions as a mediator in the interplay between Nef and EXOC (the exocyst complex). This mediation is pivotal for Nef's capacity to inhibit TCR-induced actin remodeling, a process fundamental to facilitating viral dissemination and immune evasion (30). The association of Nef with Pak2 perturbs actin cytoskeletal function and impairs the stabilization of cell polarity of HIV-1 infected T cells; furthermore, when subjected to immune pressure mediated by cytotoxic T lymphocytes (CTLs), the Nef-induced inefficient migration maintains HIV-1 persistence (31).

Nef-associated factor 1 (Naf1) is discovered by yeast two-hybrid screening and pull-down assay using HIV-1 Nef as the bait (32). Naf1 has multiple cellular regulatory functions via activating signal pathways (33). It contains multiple tyrosine residues that could be phosphorylated by SFKs. P-selectin recruits SFKs to phosphorylate Naf1 at the Tyrosine-552, and the phosphorylated Naf1 then stimulates PI3K signaling to activate leukocyte integrins (34). In the context of HIV-1 infection, Naf1 can be incorporated into HIV-1 virions and interacts with the matrix protein (35). We and others have found that Naf1 is a nucleo-cytoplasmic shuttling protein (35, 36). The cytosol-located Naf1 suppresses NF-κB signaling and consequently blocks HIV-1-LTR-driven gene expression and maintains viral latency (37), whereas the nucleus-located Naf1 can associate with CRM1 (chromosome region maintenance, also known as exportin (i) to promote nuclear export of unspliced HIV-1 *gag* mRNA, leading to the accumulated Gag production to facilitate viral assembly and release (36). Intriguingly, Nef shows antagonism against Naf1 in modulating gene expression. Naf1 overexpression increases cell surface CD4 expression, which can be down-regulated by Nef (32). In this study, we investigated how the interaction between Nef and Naf1 regulates HIV replication.

## RESULTS

### Nef activates HIV-1 proviral transcription by recruiting SFKs to stimulate the PI3K/AKT/mTOCR1 cellular pathway

**Fig 1:**
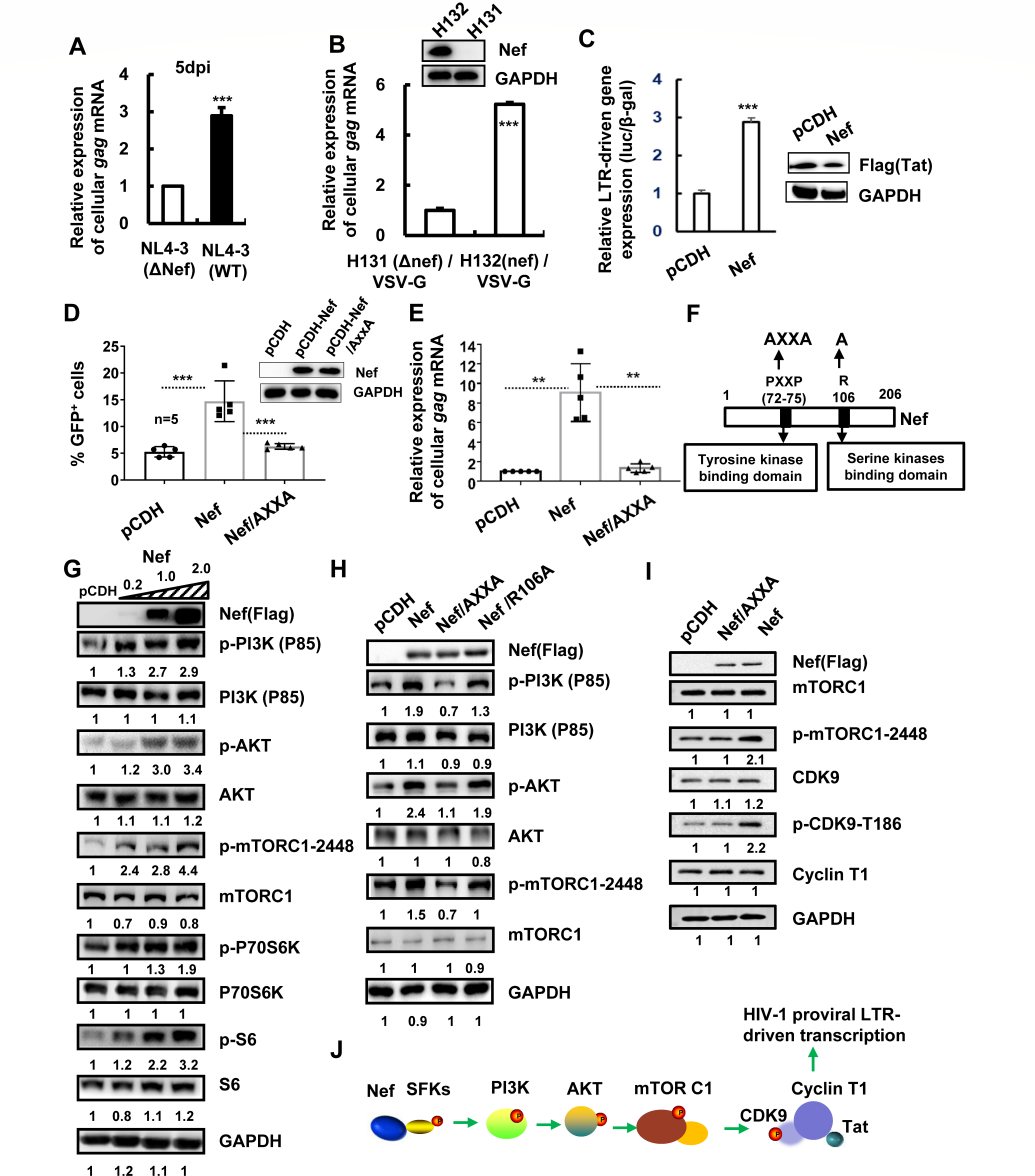
Nef recruits Src and promotes HIV-1 proviral DNA transcription by stimulating PI3K/AKT/mTOCR1 pathways. (**A, B**) Nef promotes HIV-1 replication. PHA-P-activated CD4^+^ T cells (10^6^ cells) were infected with replication-competent HIV-1_NL4-3_ (WT) or Nef-deficient (ΔNef) mutant virus (50 ng p24^gag^ amounts of viruses) (**A**), or with pseudotyped single-cycle infectious HIV-H131(ΔNef)/VSV-G or HIV-H132 (Nef)/VSV-G (5 ng p24^gag^ amounts of viruses) (**B**), for 5 days. Viral replication was detected by measuring the production of cell-associated *gag* mRNA. (**C**) Nef increases LTR-driven gene expression. Nef-expressing plasmid pCDH-CMV-MCS-EF1-Puro-Nef, HIV-1-*tat* expressing plasmid pRK-Flag/tat, and a luciferase reporter driven by the full-length LTR promoter derived from HIV-1_NL4-3_ were co-transfected into HEK293T cells, and β-Gal-expressing vector was used to normalize transfection efficiency. At 24 h post-transfection, cells were harvested, and the reporter gene expressions were assessed. (**D, E**) Nef promotes the transcription of HIV-1 proviral DNA. C11 cells were transduced with lentiviruses (5 ng p24^gag^ amounts of viruses) containing HIV-1 Nef- or mutant-expressing plasmid or vector control for 2 days; the transcription of HIV-1 proviral DNA was measured by detecting GFP expression (**D**) or measuring the production of cell-associated *gag* mRNA (**E**). (**F**) The illumination of Nef motif and mutants. (**G through J**) Nef activates the PI3K/AKT/mTORC1 pathway. C11 cells were transfected with pCDH-CMV-MCS-EF1-Puro-Nef (0.2, 1, or 2 ng plasmids in “G”; 1 ng plasmids in “H” and “I"), mutant-expressing plasmid, or a vector control for 2 days. The expression and phosphorylation of PI3K p85 subunit, AKT, mTORC1, P70S6K S6, and CDK9 were detected by western blotting. One representative from at least five repeats is shown. The Image J software was used to calculate the gray intensity of western blotting strips, and the relative values were labeled below (**G through I**). **P < 0.01 and ***P < 0.001 are considered significant differences determined by an unpaired t test.

Nef can enhance HIV-1 replication (38), and we first confirmed this point in CD4^+^ T cells. PHA-P-stimulated primary CD4^+^ T cells were infected with replication-competent HIV-1_NL4-3_ (WT) or Nef-deficient (ΔNef) mutant virus (Fig. 1A) or with pseudotyped HIV-H131(ΔNef)/VSV-G or HIV-H132 (Nef)/VSV-G (Fig. 1B) for 5 days. The presence of Nef significantly promoted the production of *gag* mRNAs (Fig. 1A and B). Nef-promoted Tat-induced LTR activity, evidenced by results from HEK293T cells co-transfected with Nef-expressing plasmid pCDH-CMV-MCS-EF1-Puro-Nef, Tat-expressing plasmid pRK-Flag/tat, and a luciferase reporter driven by the full-length LTR promoter derived from HIV-1_NL4-3_ (Fig. 1C).

We extended to explore Nef’s role in activating the transcription of integrated proviral DNA. The HIV-1 latently infected Jurkat T-cell clone (C11) that harbors an HIV-1 proviral DNA with insertion of a green fluorescent protein (GFP) encoding gene was used (37, 39–41). C11 cells were transduced with lentiviruses containing HIV-1 Nef-expressing plasmid pCDH-CMV-MCS-EF1-Puro-Nef or vector control. The transcription of HIV-1 proviral DNA was quantified by detecting GFP expression and cellular *gag* mRNA production. Nef expression increased GFP^+^ cells (Fig. 1D) and enhanced the production of *gag* mRNAs (Fig. 1E).

Nef exerts its functions by associating with host proteins. SFKs are the major non-receptor tyrosine kinases expressed in multiple types of human cells (42). The intact ^72^P(proline) XXP^75^ motif of Nef that mediates binding to the SH3 domain of SFKs is required for Nef's ability to downregulate MHC-1 and enhance virion infectivity (43). R106 is at the N-terminal end of α-helix 2 of Nef, and the R106A mutant is fully defective for Pak2 activation (44, 45). To examine whether SFK activation mediates Nef-induced proviral DNA transcription, we constructed a Nef mutant, in which the proline at positions 72 and 75 in the ^72^PXXP^75^ motif was doubly mutated to alanine (Nef/AXXA) on the plasmid of pCDH-CMV-MCS-EF1-Puro-Nef (Fig. 1F), according to the report (46). The Nef/AXXA mutant abolished the ability to activate the transcription of HIV-1 proviral DNA (Fig. 1D and E), suggesting the requirement of association with SFKs for Nef activating the transcription of HIV-1 proviral DNA.

Next, we investigated the downstream cellular pathways by which Nef activates the transcription of HIV-1 proviral DNA. Nef has previously been reported to assemble SFKs/PI3K cascade to downregulate cell-surface MHC-1, increase HIV-1 production, and accelerate pathogenesis (28, 29, 47); Nef synergies with KSHV (Kaposi’s sarcoma-associated herpesvirus) oncoprotein K1 to accelerate K1-induced angiogenesis by activating PI3K/AKT/mTOR signaling (47). To investigate whether PI3K/AKT and the downstream pathways are responsible for Nef-induced transcription of HIV-1 proviral DNA, we over-expressed Nef protein in C11 cells and performed western blotting by using specific antibodies to detect the activation of this cellular pathway. Nef expression increased the phosphorylation of PI3K p85 subunit, AKT, mTORC1 (p-mTOR-2448), and the downstream proteins, P70S6K and S6, in a dose-dependent manner (Fig. 1G), suggesting Nef’s activation of the PI3K/AKT/mTORC1 pathway. The overexpression of Nef/AXXA mutant that loses the capacity of recruiting SKFs abolished Nef-induced activation of the PI3K/AKT/mTORC1 pathway (Fig. 1H), demonstrating the requirement to recruit SFKs for Nef-mediated activation of this cellular pathway. As the control, the mutant R106A, with the defect in activating Pak2 serine/threonine kinase (30, 48, 49), did not reduce Nef-induced activation of this cellular pathway (Fig. 1H).

The mTORC1-mediated pathway has been reported to induce the phosphorylation of CDK9 (cell cycle-dependent kinase 9) to consequently activate HIV-1 proviral DNA transcription (50). We therefore investigated whether the mTORC1/CDK9 axis was engaged in Nef-mediated HIV-1 proviral DNA transcription. Nef overexpression increased the phosphorylation of Ser-2448 of mTORC1 (p-mTOR-2448) and Thr-186 of CDK9 (p-CDK9-186), but overexpression of Nef/AXXA has no effect on the phosphorylation of either mTORC1 or CDK9 (Fig. 1I). These results demonstrate that Nef recruits SFKs to activate the mTORC1/CDK9 axis.

Taken together, these data demonstrate that Nef activates HIV-1 proviral DNA transcription via recruiting SFKs to activate the PI3K/AKT/mTOCR1 pathway (Fig. 1J).

### Naf1 is required for Nef’s function, although Naf1 is a suppressive factor of the PI3K/AKT/mTOCR1 cellular pathway

**Fig 2:**
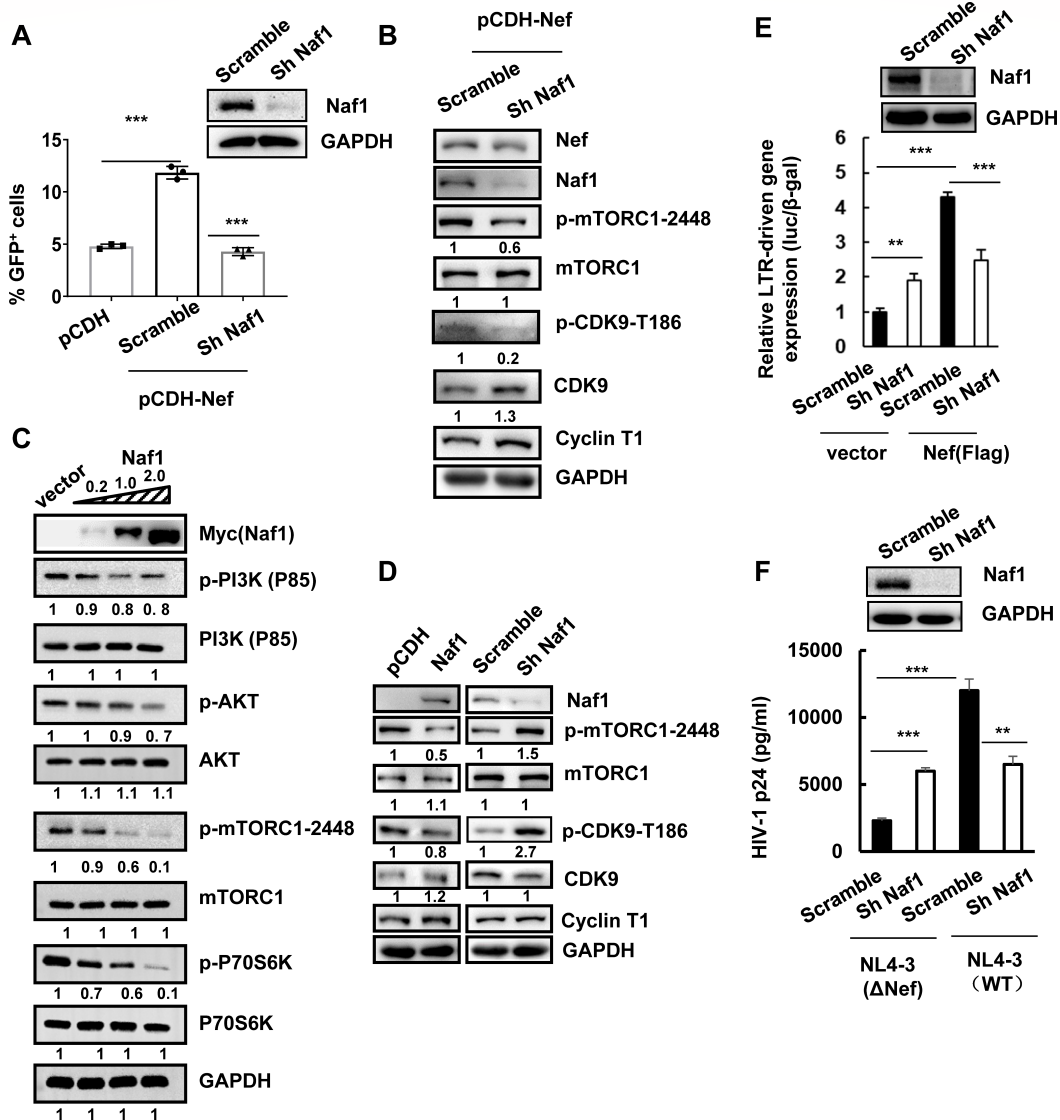
Naf1 is required for Nef-induced transcription of HIV proviral DNA. (**A**) C11 cells were transduced with lentiviruses containing Naf1 shRNA or the scramble control for 48 h and further transduced with pCDH-CMV-MCS-EF1-Puro-Nef or a vector control for an additional 48 h. HIV proviral DNA transcription was measured by detecting GFP expression, and the protein expression and Nef-induced phosphorylation of PI3K p85 subunit, AKT, mTORC1, and CDK9 were detected by western blotting (B). (**C–E**) Naf1 suppresses the PI3K/AKT/mTOCR1 pathway. C11 cells were transfected with pCMV-Tag3B-myc-Naf1 (0.2, 1, or 2 ng plasmids) or a vector control (**C**), or transduced with lentiviruses containing Naf1 shRNA or the scramble control (**D**), for 48 h. Western blotting was performed to detect the protein expressions and the phosphorylation levels of p85, AKT, mTORC1, P70S6K, and CDK9 (**C, D**). (**E**) LTR-driven gene expression. HEK293T cells were transduced with lentiviruses containing Naf1 shRNA or scramble control for 48 h, then were further transfected with pCDH-CMV-MCS-EF1-Puro-Nef, pRK-Flag/tat, and a luciferase reporter driven by HIV-1_NL4-3_-LTR for 24 h, and the reporter gene expressions were assessed. (**F**) Viral replication. PHA-P-activated CD4^+^ T cells (10^6^ cells) were transduced with lentiviruses containing Naf1 shRNA or scramble control for 2 days, then were further infected with HIV-1_NL4-3_ (WT) or Nef-deficient (ΔNef) mutant virus (50 ng p24^gag^ amounts of viruses) for 5 days. Viral replication was detected by measuring the p24^gag^ in cell supernatant. One representative from four repeats is shown. The Image J software was used to calculate the gray intensity of western blotting strips, and the relative values were labeled below (**B–D**). **P < 0.01 and ***P < 0.001 are considered significant differences determined by an unpaired t test.

Having demonstrated the role of Nef in activating HIV-1 proviral DNA transcription, next, we examined which Nef-interacting proteins are involved in this process. Naf1 was identified in association with HIV-1 Nef and ubiquitously expressed in human tissues, especially in peripheral blood lymphocytes (32). We found that Nef-induced transcription of HIV proviral DNA depended on Naf1 expression, as Naf1 knockdown with specific shRNA completely abolished Nef-induced HIV-1 proviral DNA transcription in C11 cells (Fig. 2A). In the context of cellular signaling, Naf1 knockdown impaired Nef-induced phosphorylation of mTORC1 and CDK9 (Fig. 2B).

However, we have previously found that Naf1 could inhibit HIV-1 LTR-driven transcription and maintain viral latency (37). To figure out the seeming contradiction for Naf1-modulating HIV transcription, we investigated the effect of Naf1 on the PI3K/AKT/mTOCR1 pathway. C11 cells were transfected with Naf1-expressing plasmid pCMV-Tag3B-myc-Naf1. We found that the overexpression of Naf1 inhibited the phosphorylation of PI3K p85 subunit, AKT, mTORC1, and the downstream P70S6K in a dose-dependent manner (Fig. 2C). When tracking the downstream mTORC1/CDK9 signaling, Naf1 overexpression inhibited the phosphorylation of mTORC1 and CDK9 (Fig. 2D), and the knockdown of Naf1 with lentiviruses containing specific shRNA enhanced the phosphorylation of both mTORC1 and CDK9 (Fig. 2D). These data suggested that Naf1 was a suppressive factor of the PI3K/AKT/mTOCR1 pathway.

In keeping with our previous finding (37), in the absence of Nef, Naf1 knocking down increased LTR-driven transcription (Fig. 2E) and enhanced viral replication in PHA-P-stimulated CD4^+^ T cells (Fig. 2F). However, in the presence of Nef, Naf1 knocking down compromised the restriction of viral replication, and in the meantime, Nef-promoted LTR transcription and viral replication disappeared (Fig. 2E and F).

Taken together, these data demonstrate that Naf1 is required for Nef-promoting LTR transcription, although Naf1 is a suppressive factor of the PI3K/AKT/mTOCR1 pathway.

### Nef recruits Src kinase to phosphorylate Naf1

**Fig 3:**
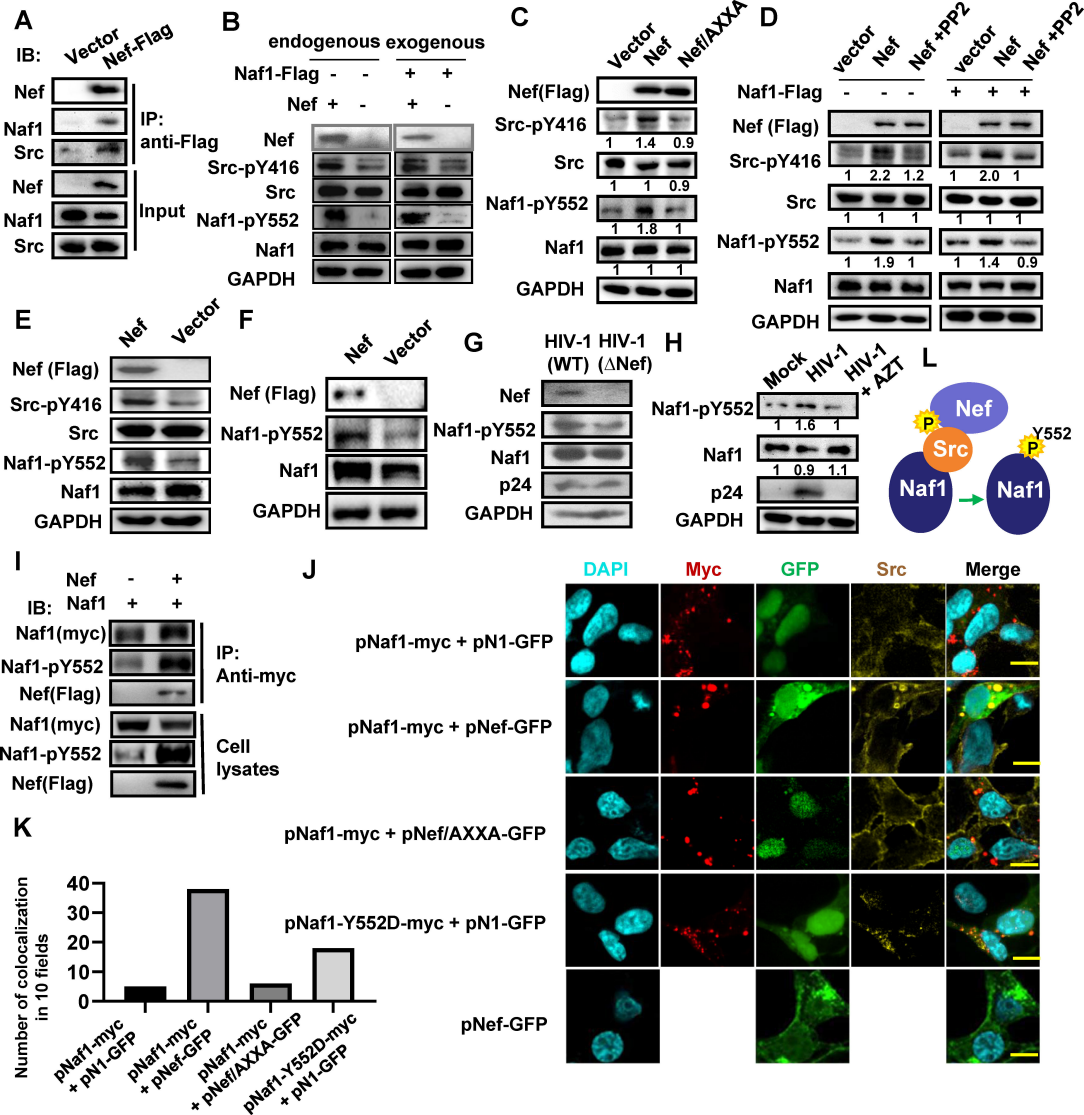
Nef recruits Src kinase to phosphorylate Naf1. (**A**) Naf1 associates with Nef and Src. HEK293T cells were transfected with pCDH-CMV-MCS-EF1-Puro-Nef with a Flag tag at the carboxyl-terminus or a vector control for 48 h. The whole-cell lysates were prepared, and immunoprecipitation was performed with anti-Flag antibody, and Naf1 and Src were immunoblotted with specific antibodies. (**B–D**) Nef activates Src to phosphorylate Naf1. HEK293T cells were transfected with Nef or AXXA mutant-expressing plasmid pCDH-CMV-MCS-EF1-Puro-Nef, with or without co-transfection of Naf1-expressing plasmid pCMV-Tag3B-myc-Naf1(B, **D**), for 48 h, and some samples were treated with PP2 (10 µM) (**D**). Protein expression and phosphorylation of Src-Y416 and Naf1-Y552 were detected by Western blotting with specific antibodies. (**E, F**) Nef phosphorylates Naf1 in CD4^+^ T cells. C11 cells (**E**) or PHA-P-activated CD4^+^ T cells (**F**) were transfected with pCDH-CMV-MCS-EF1-Puro-Nef or vector for 48 h (**E**) or 5 days (**F**), and protein expression and phosphorylation of Src-Y416 and Naf1-Y552 were detected by western blotting. (**G, H**) HIV-1 infection induces Naf1 phosphorylation. PHA-P-activated CD4^+^ T cells were infected with HIV-1_NL4-3_ (WT) or ΔNef mutant virus for 5 days. AZT (20 ng/mL) was added in some samples (**H**), and the phosphorylation of Naf1 Y552 and p24^gag^ expression were assessed by Western blotting (**H**). (**I**) Phosphorylated Naf1-Y552 associates with Nef. HEK293T cells were co-transfected with Naf1-expressing plasmid pCMV-Tag3B-myc-Naf1 and Nef-expressing plasmid pCDH-CMV-MCS-EF1-Puro-Nef with a Flag tag at the carboxyl-terminus or vector, for 48 h. The whole-cell lysates were prepared, and immunoprecipitation was performed with specific antibodies. One representative from four repeats is shown. (**J**) Immunofluorescence experiment to observe Nef-recruiting Src kinase to colocalize with Naf1. HEK293T cells were co-transfected with pCMV-Tag3B-myc-Naf1 or mutants and pNef-GFP, mutants of vector pN1-GFP, for 24 h, and immunostaining was performed with specific antibodies. Bar, 5 µm. (**K**) The colocalization of Src with Naf1 or Nef was calculated. (**L**) An illustration of the interactions between Nef, Naf1, and Src.

Next, we investigated why Nef depends on the Naf1 suppressor for promoting proviral DNA transcription. Because we have above found that Nef recruited tyrosine kinase SFKs to activate proviral transcription, we investigated the potential association of Naf1 with Nef/SFKs. SFKs include Hck, Lyn, Fyn, Src, Lck, and the association of Naf1 with Src has been previously reported (34). HEK293T cells were transfected with a Nef-expressing plasmid pCDH-CMV-MCS-EF1-Puro-Nef with a Flag tag at the carboxyl-terminus or a vector control. The cell lysates were immunoprecipitated with anti-Flag antibodies and immunoblotted with specific antibodies against Naf1 or Src, respectively. Nef showed co-immunoprecipitation with Naf1 and Src (Fig. 3A), indicating the association of Naf1, Nef, and Src.

Activated-SFKs interact and modify the downstream proteins by phosphorylation of tyrosine residues (51). Naf1 contains multiple tyrosine residues, among them, the phosphorylation at 552-tyrosine residue (Y552) could recruit the PI3K kinase p85-p110delta heterodimer to activate this pathway (34). To investigate whether the Y552 of Naf1 could be phosphorylated by Nef, HEK293T cells were transfected with Nef-expressing plasmid pCDH-CMV-MCS-EF1-Puro-Nef with or without co-transfection of Naf1-expressing plasmid pCMV-Tag3B-myc-Naf1. Src activation and Naf1-Y552 phosphorylation were detected by western blotting. The specific antibodies against phosphorylated Naf1 at Y552 were used for detection (34). SFKs undergo intermolecular auto-phosphorylation commonly on Try-416 to retain the catalytic pocket into their fully active conformation (52). The overexpression of Nef derived from HIV-1 NL_4-3_ induced the phosphorylation of endogenous Src at Try-416, and simultaneously, greatly elevated phosphorylation of both endogenous and transfected Naf1 at Y552 (Fig. 3B and D). As expected, the Nef AXXA mutant that cannot recruit Src did not induce Src phosphorylation and abolished Nef-induced Naf1 phosphorylation (Fig. 3C). Moreover, the addition of a selective SFK inhibitor PP2 suppressed Src phosphorylation and the Y552 phosphorylation of both endogenous and transfected Naf1 (Fig. 3D). The transfection of C11 cells (Fig. 3E) or PHA-P activated primary CD4^+^ T cells (Fig. 3F), with pCDH-CMV-MCS-EF1-Puro-Nef, promoted phosphorylation of Naf1-Y552 and/or Src.

To evaluate the role of Nef-induced phosphorylation of Naf1-Y552 in virus infection, the PHA-P-stimulated primary CD4^+^ T cells were infected with HIV-1_NL4-3_ (WT) or Nef-deficient (ΔNef) mutant virus for 5 days. Results showed that Naf1 phosphorylation was enhanced in cells infected with WT HIV-1 compared with ΔNef mutated virus (Fig. 3G). The treatment with the reverse transcriptase inhibitor zidovudine (AZT) that blocked viral replication abolished Naf1-Y552 phosphorylation (Fig. 3H). The phosphorylated Naf1-Y552 associated with Nef, evidenced by immunoprecipitation assay in HEK293T cells, which were co-transfected with Naf1-expressing plasmid pCMV-Tag3B-myc-Naf1 and Nef-expressing plasmid pCDH-CMV-MCS-EF1-Puro-Nef with a Flag tag at the carboxyl-terminus (Fig. 3I).

The immunofluorescence experiment was used to reinforce this finding of Nef recruiting Src kinase to colocalize with Naf1. HEK293T cells were co-transfected with pCMV-Tag3B-myc-Naf1 or mutants and pNef-GFP, mutants of vector pN1-GFP, for 24 h, and immunostaining was performed. Nef expression recruited Src to colocalize with Naf1, and expectedly, the Nef AXXA mutant that cannot recruit Src did not induce the colocalization, and the phosphorylation-mimic mutant Naf1-Y552D could recruit Src to colocalize with Naf1 (Fig. 3J and K).

Taken together, these data demonstrate that Nef recruits Src kinase to phosphorylate Naf1 at the 552-tyrosine residue (Fig. 3L).

### Naf1-Y552 phosphorylation activates PI3K/AKT/mTORC1 signaling and compromises Naf1's inhibition on HIV proviral DNA transcription

**Fig 4:**
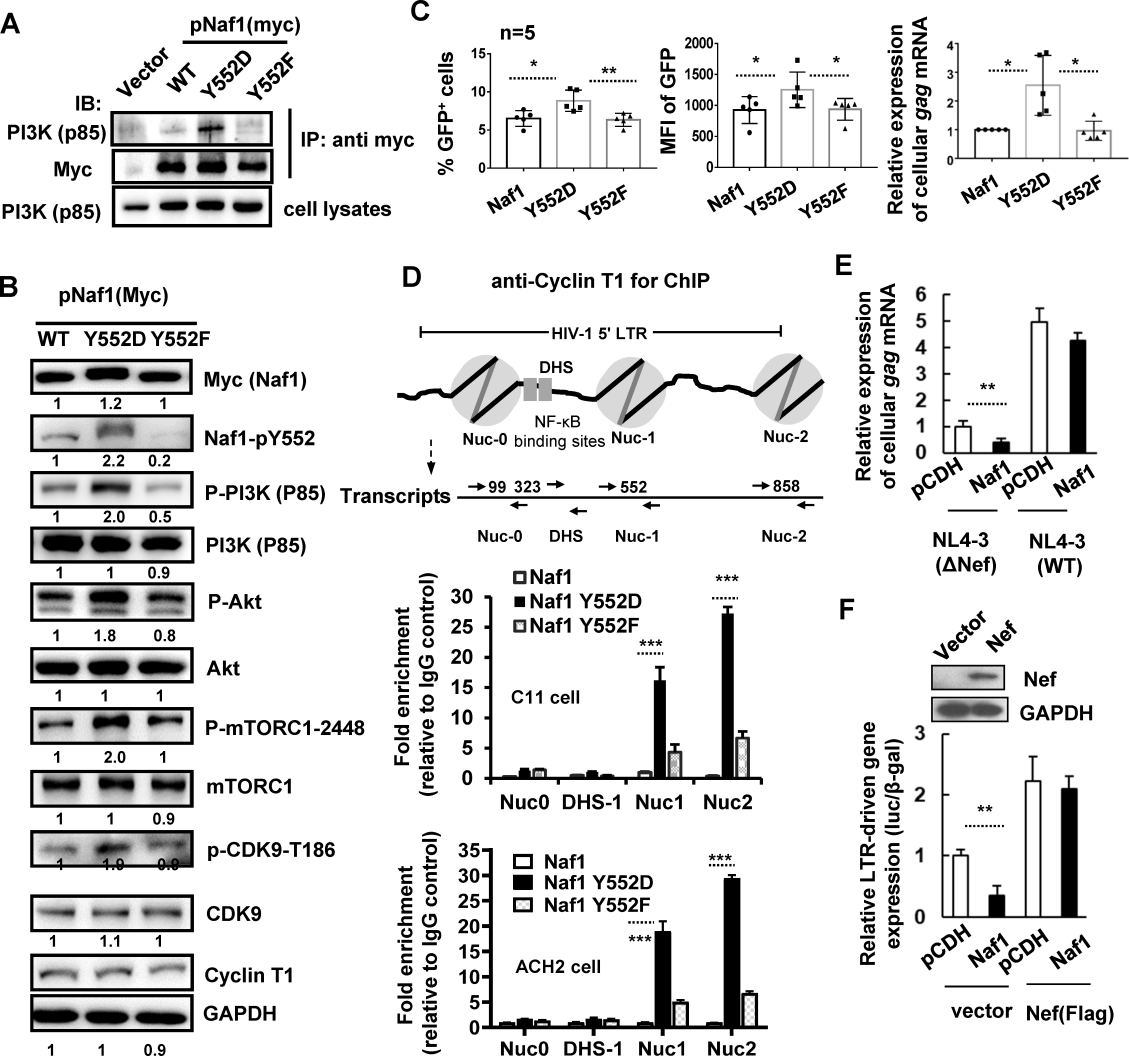
Naf1-Y552 phosphorylation activates PI3K/AKT/mTORC1 signaling and compromises Naf1's inhibitor roles in HIV proviral DNA transcription. HEK293T cells were co-transfected with pCMV-Tag3B-myc-Naf1 (or Naf1 mutant plasmids) and p85 plasmid. Immunoprecipitations were performed with anti-myc antibody, and p85 was immunoblotted with a specific antibody (**A**), and western blotting was performed to detect the expression and phosphorylation levels of Naf1, p85 subunit, AKT, mTORC1, and CDK9 (**B**). (**C**) Naf1-Y552D induces transcription of HIV proviral DNA. C11 cells were transduced with lentiviruses containing WT Naf1-expressing plasmid pCDH-CMV-MCS-EF1-Puro/Naf1, Y552D, or Y552F mutant-expressing plasmid. The transcription of HIV proviral DNA was measured either by detecting GFP expression or quantifying the production of cell-associated gag mRNA. The results from five repeats (*n* = 5) are summarized. Mean fluorescence intensity (MFI) was calculated. (**D**) The association of Cyclin T1 with HIV-1 5`-LTR in C11 or ACH2 cells was determined by a cross-linked ChIP assay. (**E**) Nef compromises Naf1's inhibitory roles of HIV-1 replication. Jurkat CD4^+^ T cells were transfected with pCMV-Tag3B-myc-Naf1 or vector and then infected with HIV-1_NL4-3_ or ΔNef mutant virus for 48 h. Viral replication was detected by measuring the production of cellular gag mRNA. (**F**) Nef compromises Naf1's inhibitory roles of LTR activation. HEK293T cells were transfected with pCMV-Tag3B-myc-Naf1, pCDH-CMV-MCS-EF1-Puro-Nef, pRK-Flag/tat, and a luciferase reporter driven by HIV-1_NL4-3_-LTR, for 24 h, and the reporter gene expressions were assessed. One representative from four repeats is shown. *P < 0.05, **P < 0.01, and ***P < 0.001 denote the significant difference determined by an unpaired t test.

We next examined whether Naf1 phosphorylation at Y552 was responsible for its functional change by regulating PI3K/AKT/mTORC1 signaling. We constructed a Naf1 phospho-mimetic mutant Y552D and an unphosphorylated mutant Y552F, in which the tyrosine at position 552 was substituted with aspartate or phenylalanine, respectively. The plasmid expressing Naf1 WT or mutants was transfected into HEK293T cells. The immunoprecipitation results showed that the phospho-mimetic Naf1 mutant Y552D displayed a strong association with the subunit p85 of PI3K, but the unphosphorylated mutant Y552F did not show the interaction with p85 (Fig. 4A). Moreover, the Naf1 Y552D mutant showed co-colocalization with Src in transfected HEK293T cells with an immunostaining assay (Fig. 3J). When tracking the cellular signaling, Naf1 Y552D, but not Y552F, enhanced the phosphorylation of PI3K subunit P85, AKT, mTORC1, and CDK9, indicating the activation of PI3K/AKT/mTORC1/CDK9 signaling (Fig. 4B).

We then evaluated whether this phosphorylated Naf1 could induce transcription of HIV proviral DNA. The C11 cells were transduced with lentiviruses containing WT Naf1-expressing plasmid pCDH-CMV-MCS-EF1-Puro/Naf1, Y552D, or Y552F mutant-expressing plasmid. The transcription of HIV proviral DNA was measured either by detecting GFP expression or quantifying the production of cell-associated *gag* mRNA. The overexpression of Naf1 phospho-mimetic mutant Y552D, but the unphosphorylated mutant Y552F, activated HIV-1 proviral transcription, featuring in the enhanced GFP expression and the promoted production of *gag* mRNA (Fig. 4C).

HIV-1 5′-LTR-driven transcription is initiated by Tat protein binding to the TAR, then recruiting the positive transcription factors including CDK-9/Cyclin T1 complex (53). Although Naf1 phosphorylation did not induce the expression of Cyclin T1, the overexpression of Naf1 phospho-mimetic mutant Y552D significantly promoted Cyclin T1 binding to the positioned nucleosomes (NUC1, NUC2) in a ChIP assay (Fig. 4D). The HIV-1 latently infected CD4^+^ CEM cell ACH2 was used to confirm the ChIP assay, and the same results have been observed (Fig. 4D).

Next, we evaluated Naf1’s modulation of viral infection in the presence of Nef. The Jurkat CD4^+^ T cells were transfected with pCMV-Tag3B-myc-Naf1 and then infected with replication-competent virus for 48 h. Naf1 transfection inhibited the infection of HIV-1 NL4-3 (∆Nef), as detected by the *gag* mRNA production, Naf1’s inhibition was abolished in the infection of HIV-1 NL4-3 containing Nef (Fig. 4E). Consistent with our previous finding (37), Naf1 overexpression inhibited LTR-driven gene expression (Fig. 4F), but the transfection of Nef abolished this inhibition (Fig. 4F). Taken together, these results demonstrate that Naf1-Y552 phosphorylation activates PI3K/AKT/mTORC1 signaling and compromises Naf1's inhibitor roles in HIV proviral DNA transcription.

## DISCUSSION

**Fig 5:**
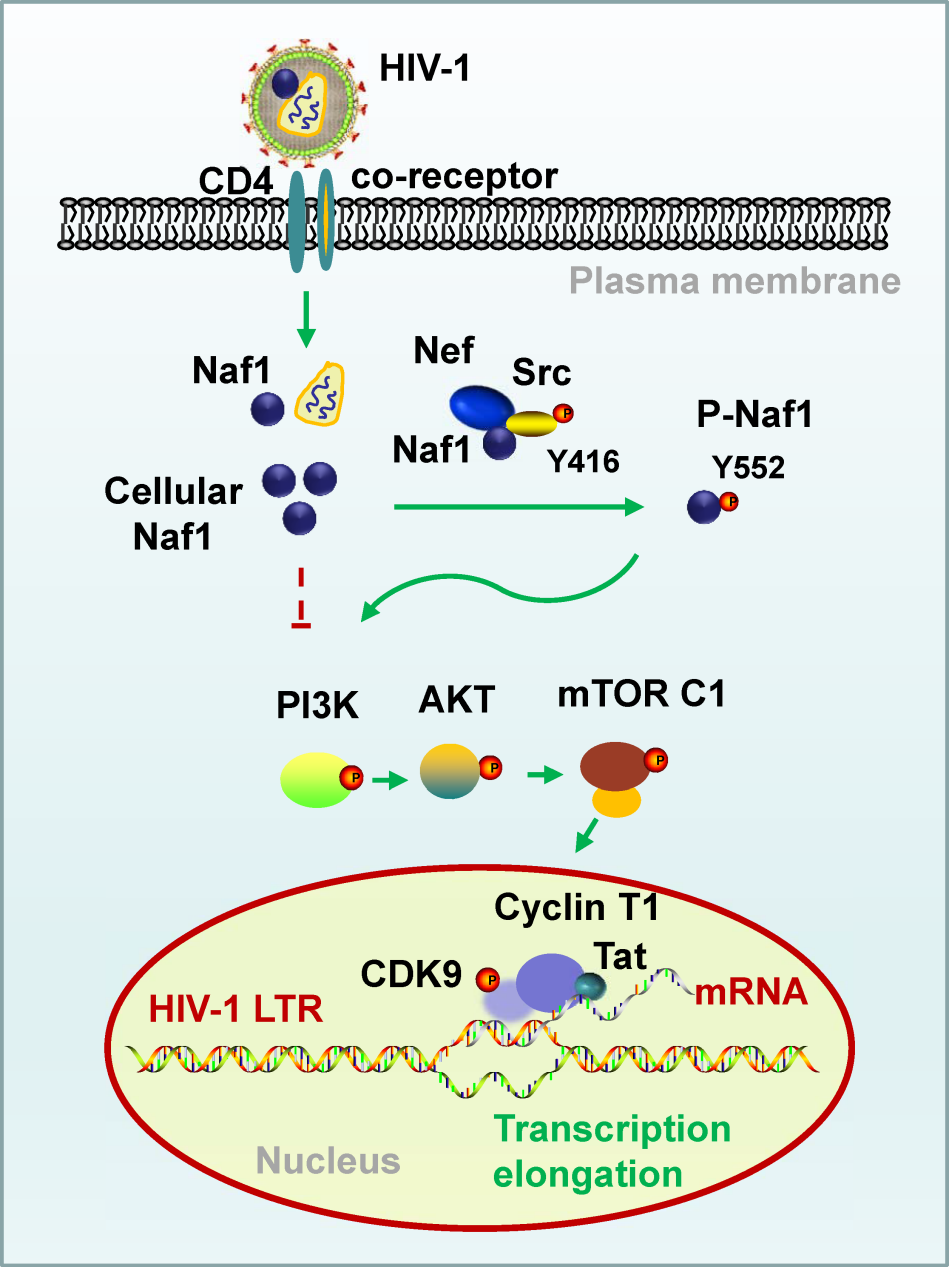
A schematic illustration of Nef promoting transcription of HIV-1 proviral DNA by recruiting Src to phosphorylate Naf1. Cellular and virion-incorporated Naf1 inhibits the PI3K/AKT/mTORC1 pathway, whereas HIV-1 Nef recruits Src to phosphorylate Naf1 at Y552, then compromises Naf1's inhibitor roles; the phosphorylated Naf1 activates PI3K/AKT/mTORC1 signaling and consequently recruits p-TEFb (the complex of CDK9 and Cyclin T1) to the LTR region to drive viral transcription from proviral DNA.

As a multifunctional pathogenic factor, Nef recruits and binds with multiple cellular proteins to manipulate cellular regulatory machineries to establish a favorable environment for viral replication and dissemination, immune evasion (1–9). Nef may modulate HIV-1 latency (54–56). The rNef (recombinant myristoylated Nef protein) from the SF2 HIV-1 strain reactivates HIV-1 latency by activating AKT in a latent model cell line 1G5 (57). The treatment of chronically infected U1 promonocytic cells with recombinant exogenous Nef proteins increases the HIV-1 replication, indicating the role of Nef-mediated reactivation of proviral DNA (58). Nef maintains the persistence of genetically intact and defective HIV-1 proviruses in effector memory CD4^+^ T cells during ART (59). However, lentiviral Nef may differentially govern the establishment of viral latency, and HIV-1 Nef promoted activation of HIV-1 infection, whereas HIV-2 Nef strongly promotes latency establishment in T cells (60). Although these studies suggest the role of Nef in modulating HIV-1 latency, the underlying cellular mechanisms are not entirely clear. In this study, we demonstrate the role of Nef in regulating HIV-1 proviral DNA transcription via activating the PI3K/AKT/mTOCR1/CDK9 cellular pathway (Fig. 5).

As a Nef-associated factor, Naf1’s role in regulating HIV-1 replication and latency has been reported (35, 37, 61). In this study, although Naf1 is a suppressor factor of the PI3K/AKT/mTOCR1 pathway, Naf1 is a prerequisite for Nef-enhanced HIV-1 proviral DNA transcription. Nef recruits Src to phosphorylate Naf1, and the phosphorylated Naf1 switches its suppression to activate the PI3K/AKT/mTORC1/CDK9 pathway and thus promotes proviral LTR-driven transcription (Fig. 5). The discordance between Nef and Naf1 in functional regulation is also reflected in other aspects. Naf1 overexpression increases cell surface CD4 expression, which can be antagonized by HIV-1 Nef (32). Nef enhances ERK2 mitogen-activated protein kinase signaling (62), but Naf1 attenuates ERK2 by blocking ERK2 nuclear import (63).

Taken together, this finding demonstrates the role of Nef in activating HIV-1 proviral DNA transcription and reveals the bilateral role of its interacting protein Naf1 in modulating HIV-1 transcription depending on the phosphorylation status. This study also provides a novel interaction mode between host restrictive factors and HIV-1 proteins in regulating viral infection.

## MATERIALS AND METHODS

### Cells

Human embryonic kidney cells (HEK293T) were cultured in Dulbecco’s modified Eagle’s medium (DMEM) (Gibco) containing 10% fetal bovine serum (FBS) (Biological Industries) and 100 U/mL penicillin and 100 ug/mL streptomycin. HIV-1 latently infected Jurkat T cells (C11 clone) were provided by Dr. Huan-Zhang Zhu (Fudan University, Shanghai, China), and the HIV-1 latently infected CD4^+^ CEM cells ACH2 were provided by Dr. Shibo Jiang (Fudan University, Shanghai, China). C11 and ACH2 cells were grown in RPMI 1640 medium supplemented with 10% fetal bovine serum (Gibco), 100 U/mL penicillin, and 100 µg/mL of streptomycin (Invitrogen) at 37°C under 5% CO_2_. Human peripheral blood mononuclear cells (PBMCs) were purchased from Shanghai Blood Center, Shanghai, China. CD4^+^ T cells were further isolated from PBMCs using anti-CD4-specific antibody-coated magnetic beads (Miltenyi Biotec, Inc.) according to the manufacturer’s instructions. CD4^+^ T cells were activated with 5 µg/mL phytohemagglutinin-P (PHA-P) (Sigma-Aldrich) for 48 h and cultured in RPMI 1640 medium with 10% fetal bovine serum (FBS) (Gibco) in the presence of recombinant interleukin-2 (IL-2) (20 IU/mL) (RD Systems).

### Plasmids and shRNA

Myc-tagged Naf1 expression plasmid pCMV-Tag3B-Naf1 was described previously (34, 37); Naf1 site-specific mutant expression plasmid was generated with the Mutant BEST Kit (TaKaRa Biotechnology), and tyrosine 552 (Y552) was mutated to aspartic acid (Y552D) or phenylalanine (Y552F), respectively, on pCMV-Tag3B-myc-Naf1 by using PCR-based mutagenesis. The sequences of Naf1 and mutants were also sub-cloned into lentiviral vector pCDH-CMV-MCS-EF1-Puro vector (Clontech) with a Flag tag at the carboxyl-terminus. The full length of *Nef* derived from HIV-1 NL_4-3_ was cloned into the pCDH-CMV-MCS-EF1-Puro lentiviral vector. The proline at positions 72 and 75 in the SH3 binding domain was doubly mutated to alanine (Nef/AXXA) on pCDH-CMV-MCS-EF1-Puro-Nef by using PCR-based mutagenesis, and the arginine at position 106 was mutated to alanine (R106A). The plasmid expressing PI3K subunit P85 was provided by Chen Wang (School of Life Science and Technology, China Pharmaceutical University, China) (34). The sequence of shRNA targeting Naf1 has been previously described (37): Naf1-sh 1842: 5′-AAT CAG AGC TCC CAA GTG ATG-3′; Scramble: 5′-TTC TCC GAA CGT GTC ACG TAT-3′. Naf1 shRNA was subcloned into the pLKO.1-puro shRNA expression lentiviral vector.

### Virus stocks

Calcium phosphate-mediated transfection of HEK293T cells was used to generate virus stock. Replication-competent HIV-1_NL4-3_ (WT) and Nef-deficient (ΔNef) mutant virus (CXCR4-tropic) were generated by transfecting pNL4-3 or pNL4-3 (∆Nef) of HIV-1 proviral vector into HEK293T cells. Supernatants harvested from transfected cells that contain viral particles were filtered and titrated with p24^gag^ capture enzyme-linked immunosorbent assay (ELISA). The HIV-1 p24^gag^-specific monoclonal antibody was provided by Dr. Yong-Tang Zheng (Kunming Institute of Zoology, Chinese Academy of Sciences, China). Viral infection was measured by p24^gag^ capture ELISA or western blotting to detect viral protein expression. Lentiviruses containing shRNAs, Naf1 (or mutants)-expressing plasmid, Nef (or mutant)-expressing plasmid were generated by calcium phosphate-mediated co-transfection of HEK293T cells with an expression plasmid of vesicular stomatitis virus G (VSV-G) protein, ∆8.9 and indicated lentiviral vectors as previously described (37, 39, 41).

### Chromatin immunoprecipitation (ChIP)

ChIP experiments have previously been described (40). C11 cells were transduced with lentiviruses containing Naf1 WT, Naf1 Y552D, or Naf1 Y552F for 3 days. C11 cells were cross-linked with 1% formaldehyde for 10 min at room temperature and quenched with 0.125 M glycine for 5 min. After lysis, nuclear extracts were separated, and chromatin was sheared by sonicator (Bioruptor UCD-200; Diagenode) for 10 min (10 s on and 10 s off) on ice to obtain DNA fragments of 200–1,000 bp in length. One percent of total sheared chromatin DNA was used as the input. Nuclear extracts were incubated with antibodies against Cyclin T1 (81464; Cell Signaling Technology) or rabbit IgG (2729, Cell Signaling Technology) at 4°C overnight. Protein G/A-labeled Dynabeads were added to each sample at 4°C for 2 h for immunoprecipitation. The immunoprecipitated DNA was analyzed by a real-time PCR (ABI Prism 7900 real-time PCR system) at 40 cycles with Thunderbird SYBR qPCR mix (Toyobo). The primers targeting the HIV LTR NUC0 (nucleosome 0), DHS (DNase hypersensitive site 1), NUC1 (nucleosome 1), and NUC2 (nucleosome 2) regions have been described previously (40): NUC0, forward, 5′-TGG ATC TAC CAC ACA CAA GG-3′, and reverse, 5′-GTA CTA ACT TGA AGC ACC ATC C-3′; DHS, forward, 5′-AAG TTT GAC AGC CTC CTA GC-3′, and reverse, 5′-CAC ACC TCC CTG GAA AGT C-3′; NUC1, forward, 5′-TCT CTG GCT AAC TAG GGA ACC-3′, and reverse, 5′-CTA AAA GGG TCT GAG GGA TCT C-3′; and NUC2, forward, 5′-AGA GAT GGG TGC GAG AGC-3′, and reverse, 5′-ATT AAC TGC GAA TCG TTC TAG C-3′.

### Immunoprecipitation and immunoblotting

Cells were lysed in radioimmunoprecipitation assay (RIPA) buffer [50 mM HEPES, pH 7.4, 150 mM NaCl, 0.5 mM EGTA, 1% protease inhibitor cocktail (Sigma), 1 mM sodium orthovanadate, 1 mM NaF, 1% (vol/vol) Triton X-100, 10% (vol/vol) glycerol] for 1 h on ice with brief vortex every 10 min. After centrifugation for 10 min at 12,000 × *g*, the lysates were incubated with the indicated antibody at 4°C overnight. Protein G/A-labeled Dynabeads were added into each sample at 4°C for 2 h for immunoprecipitation. The immunoprecipitants were separated by SDS-PAGE and analyzed by immunoblotting. For immunoblotting, cells were lysed for 1 h at 4°C in ice-cold RIPA buffer. After centrifugation for 10 min at 12,000 × *g*, the supernatant was boiled in reducing SDS sample loading buffer and analyzed by SDS-PAGE. Specific primary antibodies were used, followed by horseradish peroxidase-conjugated goat anti-mouse IgG or goat anti-rabbit IgG (Sigma) as the secondary antibodies. Five percent of total lysates was used as the input.

### Antibodies

The following antibodies were used for ChIP or immunoblotting: The specific antibodies targeting phosphorylated Naf1 at Y552 have been described (34). The antibody targeting HIV-1 Nef was provided by Dr. Yong-Hui Zheng (Michigan State University, USA). Endogenous Naf1 was detected with a mouse mAb at a dilution of 1:1,000 (34), and the following other antibodies were used: anti-Flag tag mouse (M20008; Abmart Inc., Shanghai, China), anti-GAPDH (clone 3B3) (M20006; Abmart Inc., Shanghai, China), anti-Src Rabbit mAb (2109, Cell Signaling Technology), anti-phosphorylation-Src family (Tyr416) rabbit mAb (6943, Cell Signaling Technology), anti-PI3K kinase p85 subunit (4292; Cell Signaling Technology), anti-PI3K phosphorylation p85 Tyr458/p55 Tyr 199 (4228; Cell Signaling Technology), anti-AKT (pan) (C67E7) (4691S; Cell Signaling Technology), anti-AKT phosphorylation Thr308 (D25E6) (13038; Cell Signaling Technology), anti-mTORC1 (ab32028; Abcam), anti-mTORC1 phosphorylation S2448 (ab109268; Abcam), anti-p70 S6 kinase (9202; Cell Signaling Technology), anti-p70 S6 kinase phosphor Thr389 (9205; Cell Signaling Technology), anti-S6 ribosomal protein(5G10) (2217; Cell Signaling Technology), anti-S6 ribosomal protein phosphorylation Ser235/236 (D57.2.2E) (4858; Cell Signaling Technology), anti-myc tag (ab9106; Abcam), anti-Cyclin-dependent protein kinase 9 (anti-CDK9)(2316; Cell Signaling Technology), anti-phosphorylation-CDK9 (Thr186) (2549; Cell Signaling Technology), and anti-Cyclin T1 (81464; Cell Signaling Technology).

### Immunofluorescence experiment

HEK293T cells were seeded in 12-mm-diameter coverslips and co-transfected with pCMV-Tag3B-myc-Naf1 or mutants and pNef-GFP, mutants of vector pN1-GFP (0.5 µg for each plasmid) for 24 h, and Lipofectamine 3000 (Invitrogen) was used for transfection. Cells were fixed with 3.7% formaldehyde for 20 min at room temperature and then permeabilized with 0.5% Triton X-100 in PBS for 10 min and washed three times with PBS-T (PBS + 0.1% Tween 20). For staining, cells were prior-blocked with PBS-T + 1% bovine serum albumin (BSA) for 60 min at room temperature and incubated with a primary antibody solution containing mouse anti-Myc antibody (1:1,000 dilution) and rabbit anti-Src antibodies (1:200 dilution) in PBS-T + 1% BSA for 1 h at room temperature and washed three times with PBS-T + 0.1% BSA for 5 min each. The samples were further incubated with a secondary antibody solution containing Alexa Fluor 561–conjugated donkey anti-mouse IgG antibodies (1:1,000 dilution; Invitrogen) and Alexa Fluor 647–conjugated donkey anti-rabbit IgG antibodies (1:1,000 dilution; Invitrogen) in PBS-T + 1% BSA for 5 min each at room temperature. The coverslips were stained with DAPI (4',6-diamidino-2-phenylindole) for 10 min and briefly washed with PBS. Coverslips were mounted in ProLong Gold Antifade reagent. Slides were observed using a confocal imaging system (Olympus, IXplore SpinSR).

### Statistical analysis

Statistical analysis was performed using an unpaired Student *t* test with SigmaStat 2.0 (Systat Software, San Jose, CA, USA).
